# Modeling energy consumption indexes of an industrial cement ball mill for sustainable production

**DOI:** 10.1038/s41598-025-03232-z

**Published:** 2025-05-27

**Authors:** Saeed Chehreh Chelgani, Rasoul Fatahi, Ali Pournazari, Hamid Nasiri

**Affiliations:** 1 Minerals and Metallurgical Engineering, Department of Civil, Environmental and Natural Resources Engineering, Swedish School of Mines, Luleå University of Technology, Luleå, Sweden; 2 Wallenberg Initiative Materials Science for Sustainability, Department of Civil, Environmental and Natural Resources Engineering, Swedish School of Mines, Luleå University of Technology, Luleå, Sweden; 3 School of Mining Engineering, College of Engineering, University of Tehran, Tehran, 16846-13114 Iran; 4 Shargh Cement Company, Mashhad, Iran; 5 School of Computing and Communications, Lancaster University, Lancaster, UK

**Keywords:** Cement, Ball mill, Industrial scale, Energy, Explainable artificial intelligence, Engineering, Computational science

## Abstract

The total cement energy consumption is around 5% of global industrial energy usage. In cement plants, mills consume half of this energy for dry grinding particles. However, grinding in tumbling mills is a random process, and a maximum of 5% of this energy would be directly devoted to particle size reduction. Thus, understanding interactions between operation variables and the mill energy consumption factors would be essential for sustainable cement production and green transition. Surprisingly, few investigations were conducted to study the energy consumption indexes of cement mills. Using a conscious lab “*CL*” as an advanced AI structure for industrial-scale problems could facilitate such an understanding of interactions within cement mill variables and promote controlling energy consumption for sustainable production. To fill the gap, this study developed a *CL* by examining different AI models (Random Forest, Support Vector Regression, Convolutional Neural Network, extreme gradient boosting, CatBoost, and SHapley Additive exPlanations) for modeling energy consumption indexes of a close ball mill circuit in a cement plant to address the effectiveness of operating variables. Explainable AI modeling highlighted interactions and measured the effectiveness of operating variables on mill energy consumption indexes. The airlift current and separator variables ranked the most effective operating factors on the mill energy consumption indexes. CatBoost, as an advanced AI model, showed the highest prediction accuracy for modeling (R^2^: 0.90). Such a CL model for a cement mill can be used for training operators, controlling the process, saving time and energy, reducing laboratory work, and scaling issues, and finally enhancing sustainability.

## Introduction

Cement plants use 110–120 kWh of electrical power per ton of their production (mainly for grinding). In the cement industry, the clinker grinding process accounts for approximately 40% of the electrical energy consumed^1^. Traditionally, ball mills are used in cement production to reduce raw material and clinker particle size. However, grinding in tumbling mills has a random mechanism, and only 1 to 5% of the devoted energy would be consumed to reduce the size of particles^2,3^. Around 5% of the world’s electricity production is consumed by cement plants. It was monitored that the power draw (consumed energy) range in ball mills is approximately l0 kWh/t (mill drive only) for soft, chalky limestone to 25 kWh/t for hard materials. For roller mills, the range may be 4.5–8.5 kWh/t^3^. In other words, as one of the most energy-intensive parts of the process, grinding in the cement industry needs significant process improvement to enhance the green transition and sustainable production^4^.

Controlling main motor power and elevator current is a typical tool for monitoring energy consumption in cement ball mills^5,6^. Understanding correlations between operational parameters and the mill energy index and ranking them based on their importance could help to optimize the mill power consumption. Few studies have been conducted on understanding the interaction between industrial tumbling mills’ power draw and other milling variables, where determining the mill power draw is crucial for designing, operating, and evaluating an efficient plant^7–9^. It is mainly reported that parameters such as feed rate (ton/hr), speed (rpm), length and diameter of the mill (m), and particle properties (size, density, working index, etc.) can be used for such an understanding and predicting a mill’ power draw^10,11^. Mathematical models, such as the population balance and residence time distribution, have been used to investigate possible relationships between these variables and mills’ energy consumption^12–14^. The main drawbacks of these models are that they are inconsistent, several factors of those models are not monitored in the plant, and their measurement would be quite challenging at the industrial scale. It has been well-documented that artificial intelligence (AI) systems play a key role in sustainable development^15^. AI models can solve complex problems that are frequently complicated to explain or visualize with human intelligence. Thus, using AI systems to model various mechanisms during grinding and understanding interactions could be essential in modifying energy and enhancing green production. However, there are quite a few investigations on AI modeling tumbling mills.

Tohry et al. (2020) applied Random Forest (RF) and its variable importance measurement to model the power draw of an industrial ball mill (used in a mineral processing plant). As a machine learning tool, they indicated that RF could accurately model the ball mill’s power draw and show the potential interactions within the plant’s monitored parameters^16^. As a most recent approach, the conscious lab (*CL*) concept has also been applied to model the ventilation of a cement industrial ball mill. *CL* is an AI structure that uses various explainable AI “EAI” to model interaction within industrial variables (using monitoring databases that come from plants). Using the *CL* approach, laboratory costs and scale-up challenges will be minimized, the process can be optimized, and personnel can be trained based on the process realities. The *CL* concept with different structures has successfully been applied to model complex problems^17–21^. Fatahi et al. (2021) indicated that a Boosted Neural Network with a *CL* structure could model relationships between ventilation variables and process parameters^22^. Few studies have been conducted in modeling with artificial intelligence algorithms in the context of cement and its processes. Fatahi et al. used the XGBoost algorithm on a raw material vertical roller mill in the cement industry; they found that the key factors influencing energy consumption include working pressure, input gas rate, and the superiority of XGBoost over other conventional modeling approaches was evident^17^. Another study developed a conscious-lab model using SHapley Additive exPlanations (SHAP)-XGBoost to accurately predict rotary kiln factors in a cement plant, demonstrating improved performance over typical explainable AI models^19^. A soft sensor was developed using Bayesian regularization and historical data to accurately predict raw meal fineness in a cement plant’s vertical roller mill, addressing material heterogeneity and outperforming other models^23^. Non-linear models, especially NARX networks, significantly outperformed linear models in system modeling. This led to the proposal of a neurocontroller expert system to enhance the quality, simplicity, and efficiency of the cement grinding process control^24^. A novel L/S-ConvGRU soft sensor model was developed to accurately predict cement surface area, outperforming traditional methods by effectively handling complex industrial data and improving prediction accuracy.

Nevertheless, no investigation addressed intercorrelations within the operating variables of a cement ball mill with their representative energy consumption parameters. To fill the gap, this study, with a novel approach, was structured as a *CL* by examining various machine learning methods (RF, support regression machine (SVR), Convolutional Neural Network (CNN), and extreme gradient boosting (XGB), and CatBoost (CB)). As an advanced EAI tool, SHAP and Pearson correlation were used to explore the potential interaction between model input variables and the mill energy consumption parameters (motor currents and main elevator current).

## Materials and methods

### Dataset

**Fig. 1:**
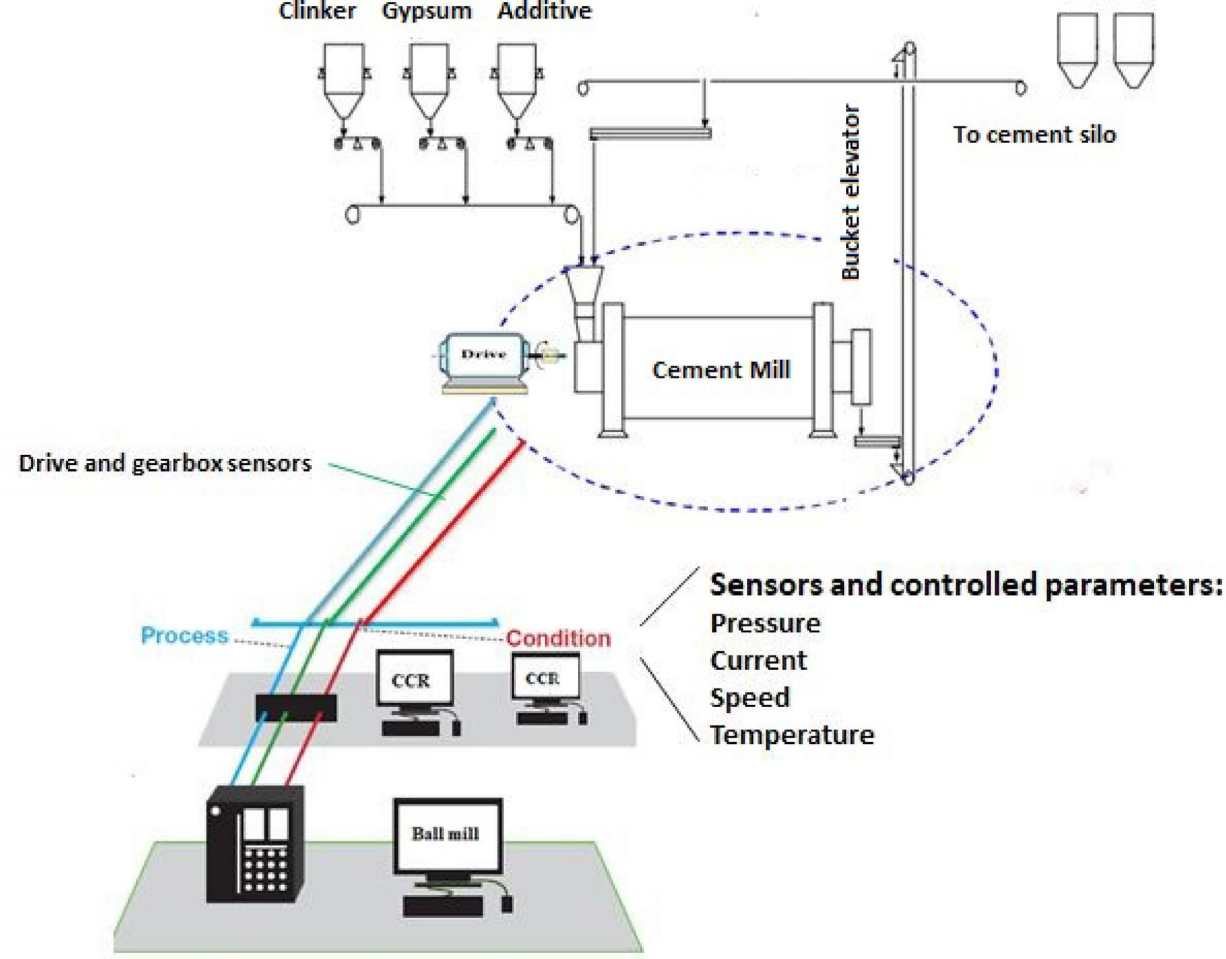
Schematic of cement ball mill circuit, and sensors for monitoring variables.

The data were provided from the monitoring section of the clinker ball mill in closed circuits from line 1 of the Ilam cement plant in Ilam (west of Iran) (Fig. 1). The plant has two cement production lines that produce 5300 TPD cement. The clinker and gypsum enter the mill by a conveyor belt to be ground into a fine powder as cement and transported by bucket elevator to cement silo storage. Almost 4% of gypsum is added during the grinding to control the setting properties of the produced cement. The ball mill has a double drive whose nominal power of each drive is 1750 KW. It also has two compartments (1sth: coarse grinding and 2nd: fine grinding), a 4.20 m diameter, and a 13 m length with 80 t/h capacity (made by PSP Company from Pˇrerov, Czechia). The mill’s rotation speeds are usually constant (15 rpm), and there is approximately a fixed one-year period of changing liners and charging the grinding media. Table 1 summarizes the critical operating parameters collected during standard operation. The data set includes two types of variables: manipulable variables and controlled variables. Manipulable variables are those set by the operator and are referred to as set points. Controlled variables include the responses of various actuators in the grinding circuit and are related to the manipulable variables. In this study, over 2600 records were recorded in the control room under different operational conditions to ensure the reliability of the data, as they are real-world industrial system data recorded with high frequency (2600 times, once per hour). The data set related to the manipulable variables, such as Separator Flap, Total Feed Rate, EP Fan Flap, and Separator Speed, were recorded as input parameters. In contrast, other variables were recorded as output parameters.

**Table 1:** The statistical description of operating variables for the clinker ball mill in the Ilam cement plant in Ilam.

Variables	Minimum	Maximum	Mean	STD
Total Feed Rate (t/h)	45.00	75.00	64.08	4.60
Air Lift Current (A)	111.00	195.00	169.35	7.02
Mill Output Temperature (°C)	60.00	151.00	116.69	8.56
Ventilation After Mill (mbar)	0.60	7.00	1.22	0.50
Ventilation Before EP Fan (mbar)	1.00	7.00	1.83	0.79
EP ΔP (mbar)	1.00	10.00	5.59	1.84
EP Fan Flap (%)	14.00	54.00	22.25	4.42
Separator Flap (%)	68.00	85.00	75.61	2.95
Separator Speed (rpm)	331.00	485.00	392.23	43.54
Separator Motor Current (A)	106.00	155.00	124.05	7.29
Separator Fan Current (A)	11.70	15.00	13.48	0.53
Main Elevator Current (A)	24.00	71.00	50.36	5.18
Motor 1 Current (A)	111.00	166.00	134.28	10.50
Motor 2 Current (A)	110.00	165.00	129.14	8.22

Air Lift Current (A) is the current draw of the motor for the airlift, which is directly related to the mill output rate of material and the total feed rate. Mill Output Temperature (^˚^C) is the outlet temperature of the mill and indicates the temperature of the cement produced. Ventilation after mill (mbar) is the ventilation after the mill. Ventilation before the electrostatic precipitator (EP) Fan (mbar) shows the suction leading to the EP fan, depending on the position of the EP fan flap. EP ΔP (mbar) is the pressure difference before and after the electro filter. EP Fan Flap (%) is the position of the EP flap that is adjusted based on the required suction. Separator Flap (%) is the position of the separator flap, which is the amount of air needed to separate the materials inside the air separator.

**Fig. 2:**
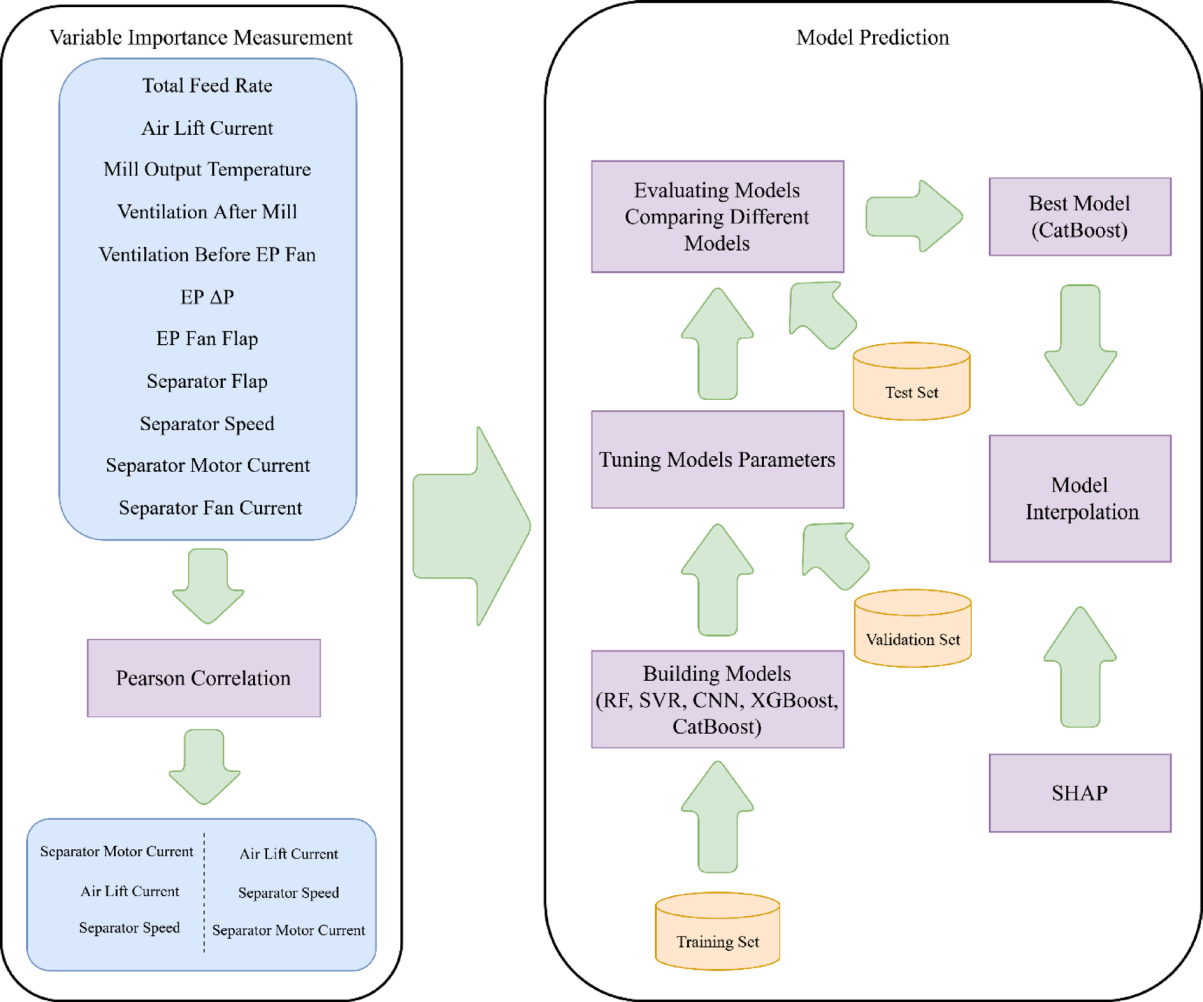
The flowchart of the proposed methodology.

Separator speed (rpm) is the rotation speed of the separator that is adjusted to achieve the required particle size distribution. The separator motor current (A) is the current draw of the separator motor. Separator fan current (A) is the current draw of the motor’s separator fan. This fan provides the air required for separation, and the position of the separator flap depends on it. Total feed rate: The aggregate tonnage of clinker and gypsum is the mill’s feed rate. All data, including power data recorded in this study regarding current intensity, are derived from the actuators (motors) performance response, which are sent online to the control room through the plant’s control system. Power data are visible to mill operators in the control room in real-time and online. In this study, during various operational conditions over the monitoring and measurement period, power data were recorded by the control room’s monitoring system. Variables were monitored hourly. After cleaning and removing missing data and outliers, 2499 cleaned data points were used for modeling. The flowchart of the methodology used to model energy consumption indexes of the industrial cement ball mill is shown in Fig. 2.

### Machine learning methods

#### SHapley Additive exPlanations (SHAP)

Shapley Additive Explanations (SHAP) is a groundbreaking method in machine learning interpretability that provides valuable insights into the predictions of complex models. Developed by Lundberg and Lee^25^, SHAP leverages Shapley values from cooperative game theory to explain the contribution of each feature to the model’s output. By computing the average marginal contributions of all feature permutations, SHAP quantifies the impact of individual features on the prediction, offering a comprehensive understanding of the model’s decision-making process^26^. This unique approach enables users to interpret black-box models and gain insights into the factors driving the predictions, enhancing transparency and trustworthiness in machine-learning applications^27^.

SHAP provides local and global explanations for model predictions, allowing users to understand the importance of each feature at the instance level and across the entire dataset. By decomposing the model’s output into contributions from individual features, SHAP offers a nuanced view of how each input variable influences the prediction, enabling users to identify critical factors and assess the model’s behavior comprehensively. The interpretability provided by SHAP enhances the transparency of machine learning models and facilitates model debugging, feature engineering, and decision-making processes^28,29^.

SHAP can provide meaningful insights into the model’s inner workings and highlight areas for optimization and refinement. By visualizing feature importance, interaction effects, and prediction explanations, SHAP empowers users to make informed decisions, validate model assumptions, and enhance the interpretability of complex machine learning systems. Mathematically speaking, the output is expressed as the summation of the input features of the model, multiplied by the corresponding SHAP values as follows:

1 $$f\left(x\right)= {\varphi }_{0}+ \sum_{i=1}^{N}{\varphi }_{i}{X}_{i}{\prime}$$

where $f$ represents the mapping function represented by the machine learning model; $N$ is the number of input features; ${\varphi }_{0}$ denotes the average of all predictions; ${\varphi }_{i}$ is the SHAP value for the *i-th* attribute; and ${X}_{i}{\prime}$ represents the coalition vector for the *i* th component, which can be calculated from the original input ${X}_{i}$ using a mapping function expressed as ${X}_{i}={h}_{x} \left({X}_{i}{\prime}\right)$^30–32^.

#### Categorical Boosting (CatBoost)

CatBoost^33^, short for Categorical Boosting, is a robust implementation of Gradient Boosted Decision Trees (GBDT) that significantly refines the traditional GBDT technique. One of the key strengths of CatBoost lies in its efficient handling of high cardinality categorical variables. Unlike popular GBDT techniques such as XGBoost and LightGBM, CatBoost utilizes a unique approach to dealing with categorical features, particularly those with high repeatability. CatBoost can effectively process categorical features without extensive memory and computational resources by employing modified target-based statistics during training. This feature sets CatBoost apart in GBDT algorithms, making it a preferred choice for datasets with sparse or infrequently occurring categorical variables^34^.

Furthermore, CatBoost introduces novel techniques such as Ordered Target statistics and Ordered Boosting to enhance the performance and accuracy of gradient boosting models. By iterating through random permutations of the dataset, CatBoost ensures that specific training examples are appropriately utilized for encoding categorical components and estimating the rate of change of the loss function. This systematic approach, as highlighted by Hancock and Khoshgoftaar^35^, not only improves the model’s ability to handle sparse or infrequently occurring categorical variables but also helps reduce variance in estimates, thereby enhancing the overall predictive capabilities of the algorithm.

Moreover, CatBoost’s emphasis on level-wise tree growth and symmetric trees is pivotal in optimizing the model’s predictive power. By employing a consistent feature selection strategy at each level of tree growth, CatBoost ensures a balanced and efficient tree structure, contributing to improved model performance. The algorithm’s capability to handle categorical features without manual preprocessing and efficiently handle high cardinality categorical variables make CatBoost a versatile and powerful tool for various machine learning tasks. The innovative features and optimizations offered by CatBoost position it as a leading choice for practitioners seeking advanced boosting algorithms with categorical feature support^33,34^. CatBoost can identify the input features’ relative importance in making predictions. The importance of a feature within a single decision tree is calculated as follows:

2 $${J}_{j}^{2 }= \frac{1}{N\sum_{n=1}^{N}{J}_{J}^{2}({T}_{n})}$$

where $N$ represents the total number of trees or iterations the algorithm is executed. The value ${J}_{j}^{2}$ indicates each feature’s overall significance or importance across the model^36^.

#### Extreme Gradient Boosting (XGBoost)

Extreme Gradient Boosting (XGBoost) is a cutting-edge machine learning algorithm that has gained widespread popularity for its exceptional performance in various predictive modeling tasks. Developed by Tianqi Chen and Carlos Guestrin^37^, XGBoost is an optimized implementation of gradient boosting that leverages parallel tree boosting to solve classification and regression problems efficiently. By incorporating regularization techniques such as L_1_ regularization (Lasso) and L_2_ regularization (Ridge), XGBoost enhances model generalization and prevents overfitting, leading to more robust and accurate predictions^38–41^. The algorithm’s ability to handle large datasets, high-dimensional feature spaces, and diverse data types makes it a primary choice for data scientists and machine learning practitioners across different industries.

XGBoost is highly stable and proficient at effectively managing outliers and noisy data, which is one of its notable advantages. XGBoost’s regularization methods and ensemble learning approach help mitigate the impact of outliers on the model’s performance, ensuring reliable predictions even in noisy or incomplete data. Additionally, XGBoost’s parallel processing capabilities enable efficient training and prediction on large datasets, making it well-suited for real-world applications where scalability and speed are crucial^42,43^. The algorithm’s low bias and variance and its robustness to outliers contribute to its superior performance compared to traditional machine learning methods, especially in scenarios with complex data patterns and diverse input features^39^.

Furthermore, XGBoost provides a comprehensive framework for hyperparameter tuning and model optimization, allowing users to fine-tune the algorithm’s parameters to achieve the best possible performance. Experts can optimize the model’s predictive accuracy and generalization capabilities by systematically exploring different hyperparameter configurations. Overall, XGBoost is a versatile and powerful machine learning tool that handles complex datasets, delivers high predictive accuracy, and provides valuable insights for data-driven decision-making. The objective function used by XGBoost has two main parts: a convex loss function and a regularization term as follows:

3 $$Obj\left(\theta \right)=L\left(\theta \right)+\Omega (\theta )$$

where $L\left(\cdot \right)$ is the loss function and $\Omega \left(\theta \right)=\gamma T+\frac{1}{2}\lambda {\Vert w\Vert }^{2}$ is a regularization function. The regularization function $\Omega \left(\theta \right)$ has two components—a term $\gamma T$ that penalizes the number of leaf nodes $T$ in the model, and a term $\frac{1}{2}\lambda {\Vert w\Vert }^{2}$ that penalizes the magnitude of the weights $w$ associated with each leaf node. The parameters $\gamma$ and $\lambda$ control the strength of these respective penalties, allowing the model complexity to be regulated^44,45^.

#### Random forest

Random Forest (RF) is a versatile and powerful ensemble machine learning algorithm widely used for classification and regression tasks. Developed by Leo Breiman^46^, RF is based on the concept of decision trees and operates by constructing a multitude of decision trees during the training phase. Each tree in the ensemble is built using a random subset of the features and a bootstrapped sample of the training data. This randomness helps to decorate the individual trees, leading to a diverse set of predictors that collectively form a robust and accurate model. RF’s ability to handle high-dimensional data and large feature spaces makes it a popular choice in various domains^47–49^.

RF can reduce overfitting and improve generalization performance. By aggregating the predictions of multiple decision trees, RF can mitigate the high variance often associated with individual trees, resulting in a more stable and reliable model. Additionally, RF is known for its robustness to outliers and noisy data, making it suitable for real-world applications where data quality may vary. The ensemble nature of RF also allows it to capture complex relationships in the data and make accurate predictions, even in the presence of noise and uncertainty^50,51^. Using an input feature vector $x={\left[{x}_{1},{x}_{2},\ldots ,{x}_{n}\right]}^{T}$, the RF method calculates the output $\widehat{\tau }\left(x\right)$ as follows:

4 $$\widehat{\tau }\left(x\right)=\frac{1}{B}\sum_{b=1}^{B}{\widehat{\tau }}_{b}\left(x\right)$$

where $B$ is the total number of trees and ${\widehat{\tau }}_{b}\left(x\right)$ represents the estimate given by the *b* th tree^20,52,53^.

#### Support Vector Regression

Support Vector Regression (SVR) is a powerful supervised machine learning method designed explicitly for regression problems. Like Support Vector Machines (SVM) for classification, SVR aims to minimize the generalization error bound rather than focusing solely on minimizing the training error^54^. This approach makes SVR particularly effective in handling complex nonlinear relationships between input and output variables. By mapping observations into a higher-dimensional feature space through nonlinear transformations, SVR can capture intricate patterns in the data and provide accurate predictions^55^.

One of the key advantages of SVR is its high generalization capability and robustness to outliers. Unlike traditional regression methods, SVR is not heavily influenced by outliers in the dataset, making it suitable for real-world applications where data may contain noise or anomalies. Additionally, SVR exhibits low computational complexity in high-dimensional spaces, meaning that its performance does not degrade as the dimensionality of the input space increases. This scalability makes SVR a versatile tool for efficiently handling large and complex datasets^56,57^.

Furthermore, SVR is known for its ability to obtain a global solution, which ensures the model’s stability and reliability across different datasets. By optimizing the parameters of the linear function using a regularization parameter and slack variables, SVR can effectively balance the trade-off between model complexity and prediction accuracy. This feature makes SVR a popular choice in various fields, including geosciences, engineering, and finance, where accurate predictions and robust models are essential for decision-making and problem-solving^58,59^. SVR employs the following function to address the regression problem:

5 $$f\left(x\right)=\langle w \varphi (x)\rangle +b$$

where w is the weight of the matrix, $\varphi (x)$ denotes the multidimensional space comprising the input vector x, and b represents the bias^19,60^.

#### Convolutional Neural Network

Convolutional Neural Networks (CNNs) have emerged as a powerful tool in deep learning, particularly in image processing and computer vision. These networks are designed to mimic the visual processing of the human brain, making them highly effective in tasks such as image recognition, segmentation, and classification. CNNs consist of neurons with learnable weights and biases, organized into multiple layers that enable the network to learn complex features from input data. One of the key distinguishing features of CNNs is their ability to leverage the spatial structure of data through convolutional layers, which extract features by applying filters across the input data. This process allows CNNs to capture hierarchical patterns and relationships within data^61,62^.

The architecture of a CNN typically includes convolutional layers followed by pooling layers and fully connected layers. Convolutional layers use filters to convolve over the input data, extracting features at different spatial locations. Pooling layers then downsample the feature maps, reducing the computational complexity while retaining important information. The fully connected layers at the network’s end combine the extracted features to make predictions or classifications. Through back-propagation, CNNs are trained on labeled datasets to adjust their weights and biases iteratively, optimizing their performance on specific tasks. This training process allows CNNs to learn to recognize patterns and features in the data, enabling them to generalize well to unseen examples and make accurate predictions^63,64^.

## Results and discussion

### Prediction

**Fig. 3:**
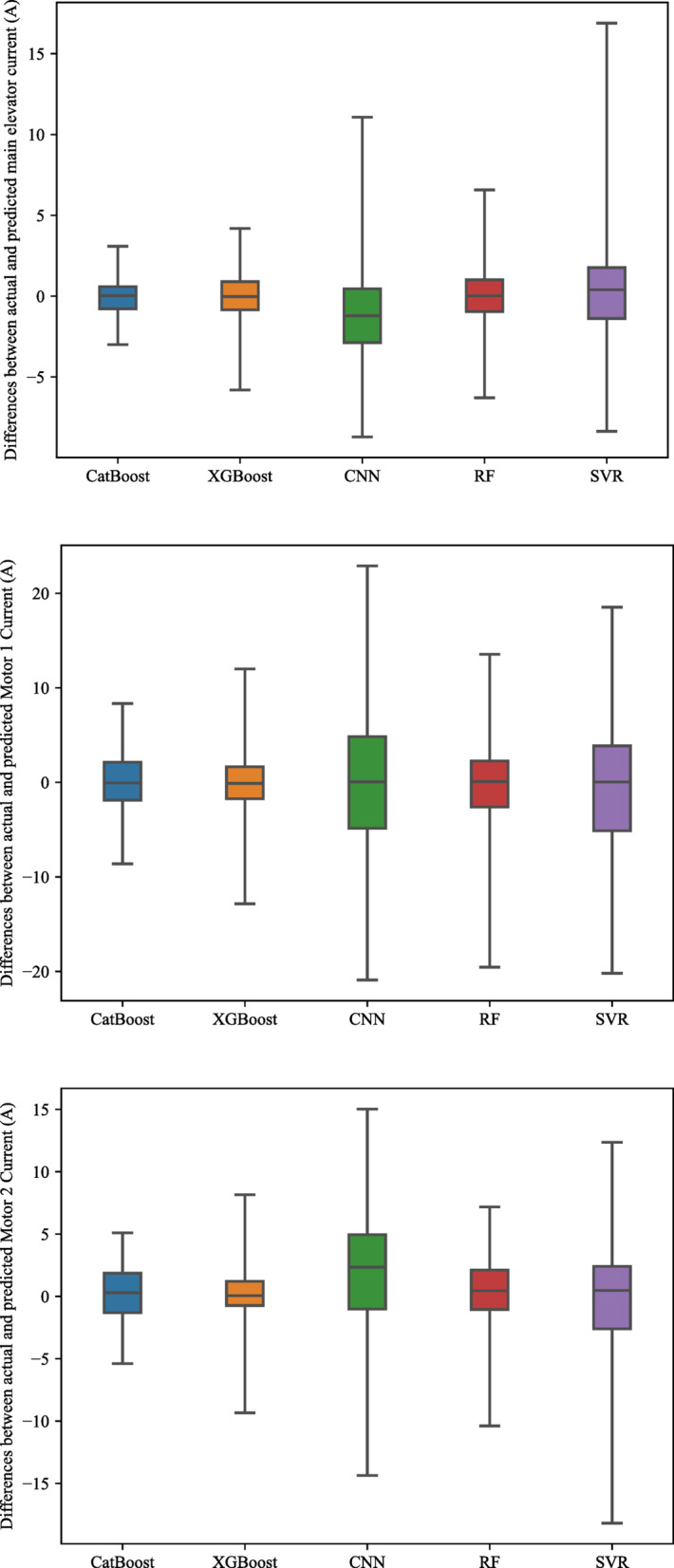
Variation between actual and predicted the cement ball mill’s energy consumption factors.

From the entire database, 70, 15, and 15% of the records were randomly split for various examined AI models’ training, validation, and testing phases (respectively). The considered machine learning models were tuned (Table 2), and their optimum hyperparameter values were adjusted through a try-and-error system using the Grid Search method. Moreover, the validation set was used to avoid overfitting and improve the models’ generalization. The results of AI models (Table 3) indicated that XGBoos and CatBoost had the highest accuracy within the examined model; however, exploring the model errors (Fig. 3) highlighted that CatBoost was the most accurate model for predicting the energy consumption factors. Moreover, to evaluate whether the superiority of CatBoost was statistically significant, a two-tailed Welch’s t-test with a significance level $\alpha =0.05$ was applied for RMSE of the test set between CatBoost and other methods. Welch’s t-test is a nonparametric univariate statistical test used when the two samples have unequal variances^65^. The last column of Table 3 shows the *p* value of the two-tailed Welch’s t-test. As can be seen, in all comparisons, the null hypothesis is clearly rejected based on the tests with a 95% confidence level (*p* value < 0.05), giving statistically significant results.

**Table 2:** The parameter settings for the predictive models.

Parameter	Main Elevator	Motor 1 Current	Motor 2 Current
CatBoost			
Learning rate	0.156	0.03	0.03
Maximum number of trees	1000 (Default)	1000 (Default)	1000 (Default)
Maximum depth of trees	6	6	6
XGBoost			
Learning rate	0.26	0.26	0.26
Maximum number of trees	29	58	35
Maximum depth of trees	18	18	26
γ	1.28	2.83	7.62
Random Forest			
Maximum number of trees	99	119	77
Support Vector Regression			
Kernel	RBF	RBF	RBF
Convolutional Neural Network			
Activation Function	ReLU	ReLU	ReLU
Training Epochs	100	100	100
Batch Size	32	32	32
Optimizer	Adaptive Moment Estimation		

*Radial basis function (RBF), **Rectified linear unit (ReLU).

**Table 3:** Outcomes of various AI models for predicting the cement ball mill’s energy consumption factors.

	Main elevator current								
	RMSE		R-squared		MAE		SSE		*p* value
	Validation	Test	Validation	Test	Validation	Test	Validation	Test	
Random Forest	1.72	1.85	0.89	0.90	1.27	1.30	640.68	605.95	1.03E−80
SVR	2.80	3.11	0.70	0.71	1.85	1.93	1,594,404.15	1,710,775.00	5.23E−85
CNN	2.96	2.96	0.67	0.67	2.60	2.91	1,280,615.65	1,386,746.94	1.10E−84
XGBoost	1.41	1.51	0.92	0.93	1.14	1.14	479.14	433.17	1.11E−77
CatBoost	1.31	1.22	0.93	0.96	1.02	0.98	361.82	300.8	**−**
Motor 1 Current									
Random Forest	4.98	4.59	0.78	0.79	3.91	3.05	5701.76	3761.20	6.82E−83
SVR	7.19	7.07	0.53	0.50	5.01	4.76	6,795,452.31	6,125,804.06	1.67E−87
CNN	8.25	8.25	0.39	0.40	5.75	5.72	8,946,815.58	8,853,701.05	1.28E−88
XGBoost	4.34	3.95	0.83	0.84	2.84	2.33	3430.27	2284.75	1.75E−79
CatBoost	4.27	3.49	0.84	0.88	2.77	2.02	3317.86	1783.59	**−**
Motor 2 Current									
Random Forest	3.05	2.69	0.87	0.88	2.24	2.15	1717.20	1572.59	4.58E−78
SVR	4.77	4.38	0.67	0.68	3.64	3.34	5,367,448.35	4,523,730.06	3.01E−85
CNN	5.70	5.70	0.54	0.56	4.35	4.33	7,658,065.86	7,213,224.21	3.20E−87
XGBoost	2.57	2.55	0.91	0.89	2.09	2.03	1406.84	1397.21	8.12E−76
CatBoost	2.63	2.37	0.90	0.91	2.11	1.89	1473.30	1194.78	–

Significant values are in bold.

According to Eq. 6, the power measured in kw directly relates to the current. Here, I is the current (A), V is the voltage (V), and cos φ is the power factor, equal to 0.88 in the cement production at the Ilam cement plant^17^.

6 $$P=\sqrt{3} IVCos\varphi$$

If the value $\sqrt{3} VCos\varphi$ is considered constant (C). Power can be expressed as in Eq. 7.

7 $$P=CI$$

As can be seen, power has a direct relationship with current. Therefore, predicting the current directly depends on the power and energy consumed.

Generally, when comparing these models, it can be mentioned that ensemble methods such as RF, XGBoost, and CatBoost combine the predictions of multiple base models, often resulting in higher accuracy than individual models. RF is a bagging model, whereas XGBoost and CatBoost employ boosting techniques, necessitating less complex feature engineering. XGBoost confronts challenges, including prediction shift and gradient boosting bias, while CatBoost mitigates prediction shift through its ordered boosting methodology. Moreover, it circumvents gradient-boosting biases by employing oblivious Decision Trees and applying a consistent splitting criterion across all tree levels^20,66^. CNNs can leverage the spatial structure of data through convolutional layers, which extract features by applying filters across the input data. This process allows CNNs to capture hierarchical patterns and complex nonlinear relationships within data; however, they are computationally intensive and require substantial resources for training. CNNs typically require large amounts of data for effective training. SVR encounters limitations due to its kernel function. Notably, empirical findings from prior studies^20,67–70^ indicated the superior performance of CatBoost over alternative machine learning techniques such as SVR, RF, and XGBoost in regression tasks.

### Intercorrelations-SHAP

**Fig. 4:**
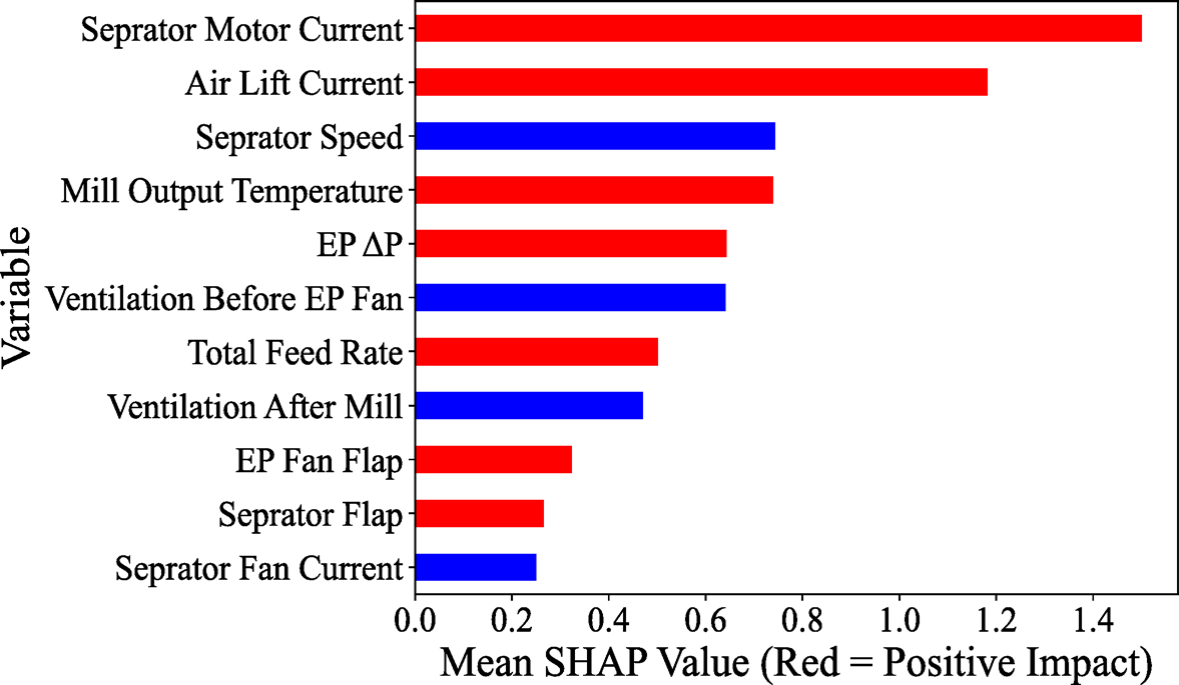
SHAP value of the operating variables for their correlation with the main elevator current.

**Fig. 5:**
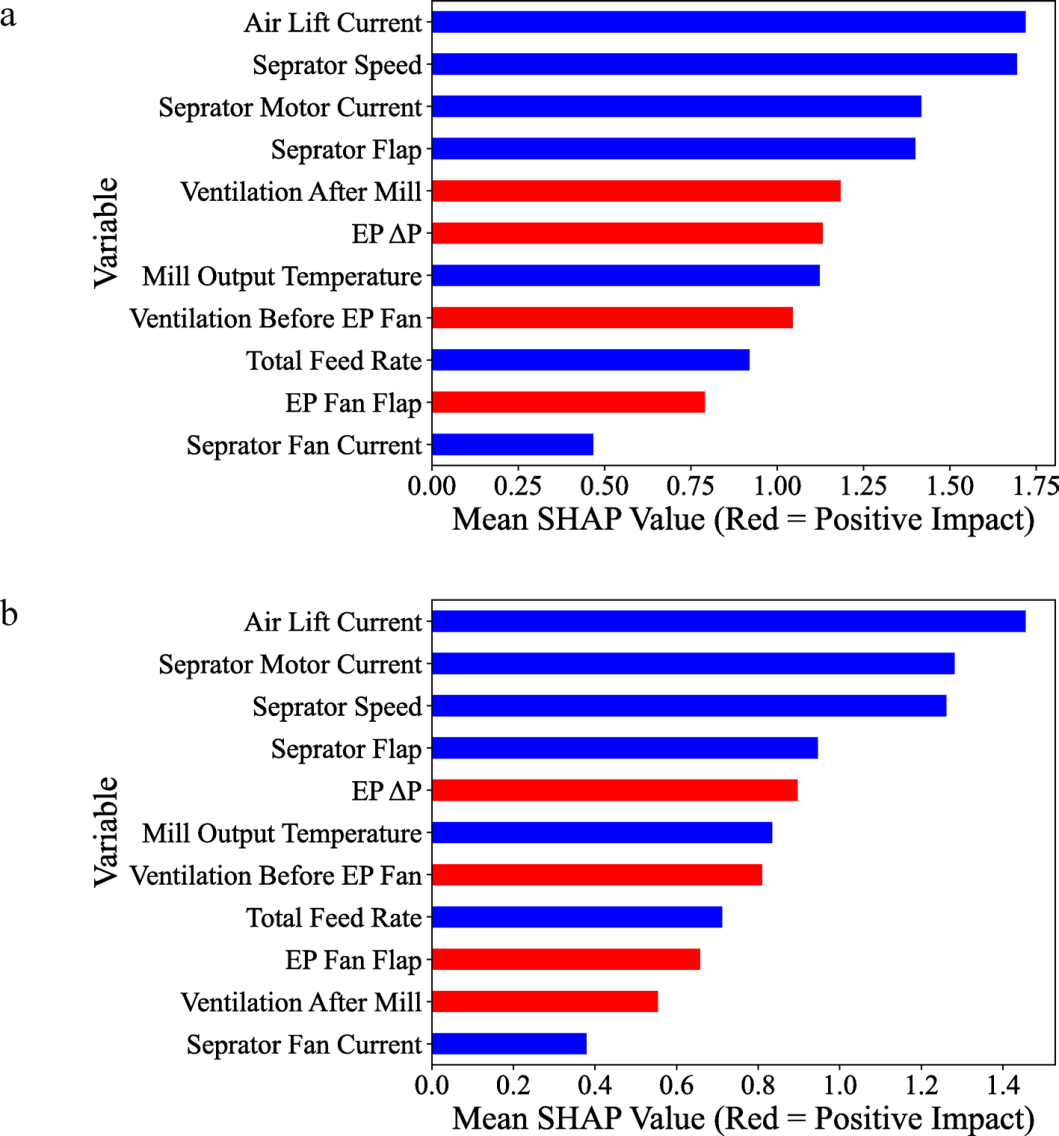
SHAP value of the operating variables for their correlation with the main motor current.

SHAP and Pearson’s correlation explored relationships between operating variables and the mill’s energy consumption representative factors modeled by CatBoost. Such an assessment helps to understand which factors have the highest importance and impact on energy consumption and the magnitude of their relationship. Based on the SHAP value (Fig. 4), the separator motor current has the highest effectiveness on the main elevator current. They showed a positive correlation. The airlift current had the highest negative interaction with the main elevator current, which decreased with the airlift current increase. The airlift current also showed the highest effectiveness on both ball mill energy consumption (Fig. 5). By increasing the airlift current, the motor current decreased (negative SHAP value on average). The magnitude of interactions between operating variables and motor currents was the same for both motors. However, the ranking of variables was slightly different.

**Fig. 6:**
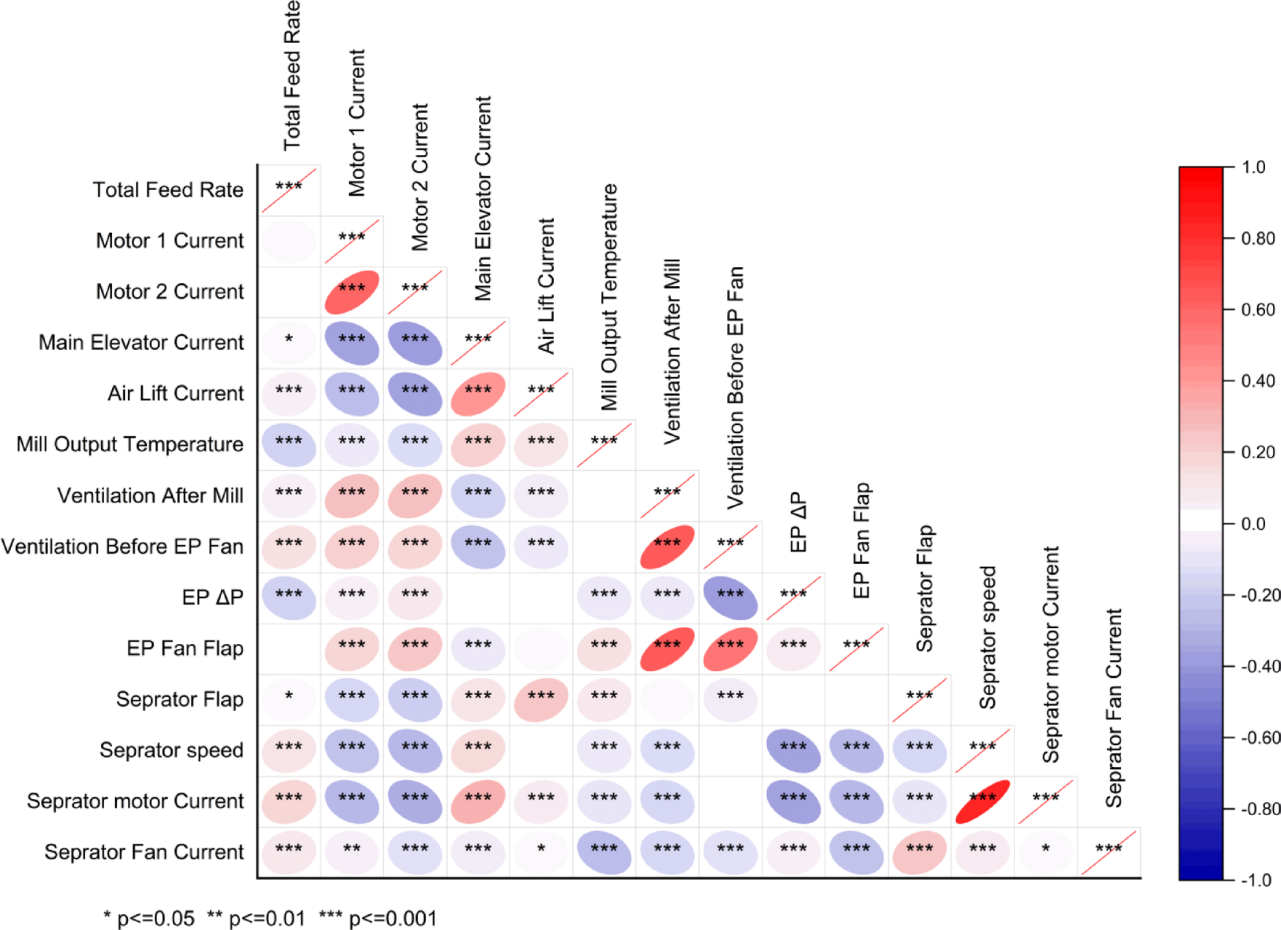
Pearson correlation between operating variables.

Exploring interactions between energy consumption indexes (Fig. 6) indicated a positive correlation (r: 0.62) between motor currents with quite a high confidence level (*p* value ≤ 0.001). Such a correlation would be the main reason that mainly all operating variables showed the same magnitude of interactions with the motor 1 and 2 currents (Fig. 5). The main elevator current also had a negative correlation (r: ~ − 0.36; *p* value ≤ 0.001) with motor currents highlighted that by increasing the motor currents, the main elevator current was decreasing.

The Pearson correlation assessment (Fig. 6) also showed several significant intercorrelations within various operating variables. For example, the ventilation after the mill demonstrated a significant positive interaction with the EP fan flap, and the separator flap had a significant positive interaction with the separator motor current. Generally, through cement grinding in a closed ball mill circuit, the mill discharge is conveyed by a bucket elevator to a separator. With control of the power (current) of the bucket elevator, the mill supply rate is adjusted to achieve the target power value. Coarse material as rejects are re-circulated to the inlet mill for further grinding, and the fine product is vented directly to the EP by airlift^71^. The airlift conveyor comprises a vertical cylindrical pressure vessel with an aeration pad at the bottom and conveying air nozzle passing upwards through the center of the pad. The quantity of material conveyed increases with vessel filling height, and blower pressure is a surrogate for material flow rate^3^. In the air separator, the separator speed and airflow at inlet and outlet separation are effective in the particle size distribution of the product because centrifuges and gravity forces carry out separation, as well as the interaction of drag forces^72^. Measuring the power or current of a mill motor is the most straightforward way to control the load of a mill^73^. The mill main motor is monitored for its current and power, as these parameters indicate the mill fill level^72^. Measuring the power or current of a mill motor is the most straightforward way to control the load of a mill^73^. The mill main motor is monitored for its current and power, as these parameters indicate the mill fill level^72^.

During grinding in a closed circuit, the relation between the current motor of the elevator and the reject material of the separator provides information on the amount of material exiting the mill outlet, and the rejected material indicates the amount of material recycled to the mill or the final product to the silo. Separator speed influences energy consumption because significant changes in the separator speed are required to improve the particle size distribution^1,74^. The cut size of the separator is the critical control parameter and is mainly controlled by separator speed^75^. The ball mill’s main motor and elevator power lift the grounded material to the separator. The elevator power is the measure of the mill load, while the main drive load is the measure of the dynamical part of the mill load due to the material’s residence inside the mill^76^. The product flow rate can be changed by changing the power of the separator fan. Hence, it increases the power drawing of the separator fan, causes an increase in the product flow rate, and affects the PSD of the product and separator speed^76^. The power consumption of the mill motor (current) indicates the operating conditions in the tube mill, such as the total material level, the condition of the grinding media and liner, system variables, and circulating load^77^. All these interactions were evaluated and ranked by SHAP-Pearson and through the conscious lab structure.

The main elevator current of the mill, located at the mill’s output, indicates the rate of the powdered product exiting the mill. As mentioned earlier, it is directly related to the power consumption of the elevator. SHAP analysis highlighted the ranking of two variables, airlift current and separator motor current, in predicting the current of the main elevator, emphasizing the significance of key energy factors in the mill grinding circuit. Through operator training in cement plants, these findings can serve as a knowledge-based strategy to help mill operators better understand and make more logical decisions during mill operations. The result of such decisions is the optimization and reduction of energy consumption.

## Conclusion

A conscious lab structure for assessing and modeling energy consumption indexes of a closed ball mill circuit for a cement plant based on monitoring operating variables was implemented as an innovative approach to understanding interactions within the process, decreasing randomness, and managing energy consumption. Considering various advanced AI models indicated that AI methods could accurately model the ball mill energy consumption factors. Catboost, as one of the most recent machine-learning methods, showed the highest accuracy and could predict the motor and elevator current with the lowest RMSE (< 3.5) and highest coefficient of determination (R^2^ < 0.90). Exploring the interactions and conducting variable importance measurements (SHAP-Pearson correlations) within operating variables to assess their effectiveness in predicting energy consumption indexes highlighted significant correlations within the energy consumption indexes, meaning monitoring and controlling could be implemented on one of them. SHAP results indicated that the airlift current, separator speed, and current were the most effective operating variables. However, certain limitations must be acknowledged. This study relied on available operational data, which, while extensive, may not fully capture all underlying complexities of the cement milling process. Potential biases in data collection, measurement errors, or missing variables could affect model generalizability. Additionally, AI models, including CatBoost and SHAP, may have challenges interpreting feature interactions for highly non-linear relationships. Future research should explore integrating additional data sources, such as sensor fusion and real-time monitoring, to enhance model robustness and improve predictions. In general, the modeling outcomes of this study indicated that SHAP-CatBoost as a robust structure could be applied to generating conscious labs dedicated to energy consumption and advantage sustainable production.

## Data Availability

The datasets used and/or analysed during the current study are available from the corresponding author on reasonable request.
